# P05.20. A study on the intra-reliability of Sasang Constitutional diagnosis by experts

**DOI:** 10.1186/1472-6882-12-S1-P380

**Published:** 2012-06-12

**Authors:** E Jang, Y Baek, K Park

**Affiliations:** 1 Korean Institute of Oriental Medicine, Daejeon, Republic of Korea

## Purpose

Sasang Constitutional Medicine (SCM) is one of the tailored traditional Korean Medicines. Experts of SCM classify human beings into four different types before treatment. It is very important for experts to diagnose Sasang constitution (SC) type correctly, as the prescriptions and methods of treatment are different according to SC types. In this study, we would like to suggest the intra-reliability of experts.

## Methods

First step, 6 experts of SC interviewed 102 subjects independently, then assigned SC types individually based on body shape, face, voice, temperament, physical and physiological symptoms of subjects. Second step, experts re-interviewed subjects 1 year later, and they finally diagnosed the 86 subjects’ SC types (Male = 39, female = 47) through mutual agreement. We analyzed intra-reliability using Cohen kappa coefficient.

## Results

Major findings were: (1) The distributions of SC types were that Taeeumin group was 42 (49%), Soeumin group 13 (15%), and Soyangin group 31 (42%) after mutual agreement. (2) The intra-reliabilities of individual experts between first and second SC diagnosis ranged from 0.380 to 0.768 of Cohen kappa coefficient. (3) When two experts diagnosed equally in first step, Cohen kappa coefficient between first diagnosis result and final one became 0.874, and if three experts diagnosed equally in first step, it became 0.893.

## Conclusion

The intra-reliability of an individual expert was not so high, but if more than two experts draw the same diagnosis result at the first step, then the reliability would be higher than that of individual expert’s diagnosis.

